# Gene/QTL discovery for Anthracnose in common bean (*Phaseolus vulgaris* L.) from North-western Himalayas

**DOI:** 10.1371/journal.pone.0191700

**Published:** 2018-02-01

**Authors:** Neeraj Choudhary, Vanya Bawa, Rajneesh Paliwal, Bikram Singh, Mohd. Ashraf Bhat, Javid Iqbal Mir, Moni Gupta, Parvaze A. Sofi, Mahendar Thudi, Rajeev K. Varshney, Reyazul Rouf Mir

**Affiliations:** 1 Division of Plant Breeding and Genetics, Sher-e-Kashmir University of Agricultural Sciences & Technology of Jammu (SKUAST-J), Chatha, Jammu, Jammu & Kashmir, India; 2 The International Institute of Tropical Agriculture (IITA), Ibadan, Oyo State, Nigeria; 3 Division of Genetics & Plant Breeding, Faculty of Agriculture (FoA), Sher-e-Kashmir University of Agricultural Sciences & Technology of Kashmir (SKUAST-K), Wadoora Campus, Sopore, Jammu & Kashmir, India; 4 Plant Biotechnology Center, Central Institute of Temperate Horticulture (CITH), Rangreth, Srinagar, Jammu & Kashmir, India; 5 Research Program Grain Legumes, International Crops Research Institute for the Semi-Arid Tropics (ICRISAT), Patancheru, Hyderabad, Telangana State, India; National Institute for Plant Genome Research, INDIA

## Abstract

Common bean (*Phaseolus vulgaris* L.) is one of the most important grain legume crops in the world. The beans grown in north-western Himalayas possess huge diversity for seed color, shape and size but are mostly susceptible to Anthracnose disease caused by seed born fungus *Colletotrichum lindemuthianum*. Dozens of QTLs/genes have been already identified for this disease in common bean world-wide. However, this is the first report of gene/QTL discovery for Anthracnose using bean germplasm from north-western Himalayas of state Jammu & Kashmir, India. A core set of 96 bean lines comprising 54 indigenous local landraces from 11 hot-spots and 42 exotic lines from 10 different countries were phenotyped at two locations (SKUAST-Jammu and Bhaderwah, Jammu) for Anthracnose resistance. The core set was also genotyped with genome-wide (91) random and trait linked SSR markers. The study of marker-trait associations (MTAs) led to the identification of 10 QTLs/genes for Anthracnose resistance. Among the 10 QTLs/genes identified, two MTAs are stable (BM45 & BM211), two MTAs (PVctt1 & BM211) are major explaining more than 20% phenotypic variation for Anthracnose and one MTA (BM211) is both stable and major. Six (06) genomic regions are reported for the first time, while as four (04) genomic regions validated the already known QTL/gene regions/clusters for Anthracnose. The major, stable and validated markers reported during the present study associated with Anthracnose resistance will prove useful in common bean molecular breeding programs aimed at enhancing Anthracnose resistance of local bean landraces grown in north-western Himalayas of state Jammu and Kashmir.

## Introduction

Common bean (*Phaseolus vulgaris* L.; 2*n* = 2*x* = 22) also known as kidney bean, French bean, dry bean, field bean etc. is one of the most precious and highly relished pulse crop used for direct human consumption globally. It belongs to family leguminosae and sub-family papilionaceae [1, 2, 3]. It is major source of calories (15%) and proteins (22%) in many developing countries throughout the world (FAO: http://faostat.fao.org/). It is also a potential source of carbohydrates (57%), minerals (0.5g/100g of edible portion), vitamin A (221 I.U.) and calcium (50 mg/100g of edible portion) [4, 5]. Common bean is grown in different parts of the world mostly for its mature dry seeds and immature tender green pods [6].

Globally, ~18 million metric tons of common beans are produced annually [7]. The world leader in production of dry bean is India, followed by Brazil and Myanmar [7]. It’s cultivation in India is mainly confined to northern hilly and plain tracts of Jammu and Kashmir (J&K), Himachal Pradesh and Uttar Pradesh as a Kharif crop and also as winter crop in some parts of Jammu and Kashmir, Maharashtra, Andhra Pradesh, Western and Eastern Ghats and North eastern plain zone, where winters are mild and frost free. In J&K, common bean is cultivated over an area of about 2000 hectares with production of about 1600 tones and yield of about 0.8 tons/ha. The beans grown in this Himalayan region of India holds greatest diversity of bean germplasm in India [8]. A preliminary study involving collection, evaluation and characterization of bean germplasm (428 lines) from this region and constitution of a core set of 96 lines was completed by us recently [8]. The study of gene pools and origin of beans from this region indicated both Mesoamerican and Andean gene pool landraces are being cultivated together in this Himalayan region. The Jammu region of the state possesses largely Mesoamerican beans, while Kashmir region possess both types of beans (Mesoamerican and Andean beans) [8].

The beans produced for this Himalayan region are preferred in the local markets across India primarily due to their taste and quality. However, yield potential of beans in this region is low compared to yield of beans in other parts of India/world. One of the main reasons for this low production/productivity is the attack by Anthracnose disease. Local landraces grown by farmers in this Himalayan region are mostly susceptible to anthracnose disease. Anthracnose is a seed-borne disease in common bean caused by *Collectotricum lindemuthianum*. This pathogen possesses high degree of pathogenic variability throughout the world including India. More than 100 races of *C*. *lindemuthianum* have been identified worldwide and number is still increasing [9, 10, 11]. Economically, anthracnose is one of the most important disease in common bean [12], since it causes huge devastation, resulting in yield losses of up to 100% in susceptible bean genotypes [13, 14, 11].

The prevalence of anthracnose disease has posed a serious threat for bean production in common bean growing areas of Himalayan state of Jammu & Kashmir, India. The most efficient way to tackle this problem is to deploy major/stable genes/QTLs through marker-assisted selection (MAS) into local common bean landraces that are mostly susceptible to anthracnose disease. Although QTLs/genes are known for this disease but there is hardly any report available about the identification of QTLs/genes for anthracnose identified from Himalayan beans genetic background. Therefore, for tackling this disease efficiently in this Himalayan region, we have collected/procured a set of 428 common bean lines from different hot-spots in state Jammu and Kashmir and from National Gene bank (NBPGR, New Delhi, India). Based on preliminary trait evaluations, qualitative trait data analysis and by keeping in view their collection sites, a set of most diverse 96 lines were selected for further studies. More details about the constitution of core set of 96 lines is available elsewhere [8]. During the present study, the core set of 96 lines was evaluated at two locations/hot-spots for anthracnose disease. The core set was also genotyped with genome-wide simple sequence repeat (SSR) markers. The data generated was used for the identification of marker-trait associations (MTAs) for anthracnose resistance. The major and stable MTAs identified during the present study will prove useful for marker-assisted breeding (MAB) programs aimed at enhancing anthracnose resistance of Himalayan beans.

## Materials and methods

### Plant material

The germplasm used in the present study comprised a diverse core set of 96 common bean landraces. The core set was constituted from a set of 428 lines/landraces based on morphological diversity of qualitative traits [8]. The 96 lines comprised of 54 local landraces from different hot-spots in state Jammu & Kashmir (8 districts of Kashmir and 3 districts of Jammu) and 42 exotic lines collected from different countries (10 different bean growing countries; S1 Table). Out of 96 lines, 45 lines belong to Meso-american gene pool while, 51 lines belongs to Andean gene pool.

### Phenotypic evaluation for anthracnose resistance

The core set of 96 common bean lines was evaluated in augmented design at two locations; Sher-e-Kashmir University of Agricultural Sciences and Technology, Chatha, Jammu (SKUAST-Jammu) and at its sub-station “Bhaderwah, Jammu”. Each genotype was grown in two rows of 3 m in length and spaced 60 cm apart. Standard agronomic practices were followed for normal crop growth at both locations. Five plants were selected in each plot for recording the disease severity data. Anthracnose disease scoring was done in field conditions at both the locations (SKUAST-Jammu and at its sub-station Bhaderwah, Jammu) after inoculation with most prevalent race of *Colletotrichum lindemuthianum* at hot-spot region “Bhaderwah” of state Jammu & Kashmir. The monosporic culture of *C*. *lindemuthianum* collected at its hot-spot “Bhaderwah” were grown on PDA media and conidia were collected in water suspension. The plants were sprayed with spore suspension and disease scoring was done at three stages of growth viz. seedling, flowering and physiological maturity. The genotypes were evaluated for anthracnose disease severity on a scale of 0–5 [15] viz 0- No disease symptoms, 1- very few small lesions covering 15% of plant surface, 2- small lesions covering 25% of plant surface, 3- enlarged lesions covering approximately 50% of plant surface, 4- severe necrosis on more than 75% of plant surface, 5- severe necrosis on more than 90% of plant surface and plant killed by pathogen (S1 Table).

### Genomic DNA extraction

The DNA extraction was done from each accession at three leaf stage using Qiagen DNeasy DNA extraction Kit (69104) using standard protocols. The concentration of DNA samples was determined using a spectrophotometer from the absorbance data of DNA sample at 260 nm (1 OD (A260) = 50 μg of doubled stranded DNA/ml). The purity of the DNA sample was determined by A260:A280 ratio (1.6–1.8 for pure DNA).

### Selection of SSR markers for genotyping

A set of 91 SSR markers were selected from earlier published reports [16, 17, 18, 19, 20, 21, 22, 23]. The criteria followed for selection of SSR included: (i) High PIC values (>0.6) in earlier published reports; (ii) Maximum number of alleles detected in earlier studies; (iii) coverage i.e., SSRs uniformly distributed on all the 11 linkage groups. Out of these 91 markers used during the present study, 59 were random genomic SSRs and 32 were carefully selected and are either EST-derived SSRs, or SSR linked to genes/QTLs for anthracnose, or other traits like Zn, Fe, yield contributing traits. The primers for SSR were synthesized on contract from Sigma Aldrich, Bangalore, India.

### SSR marker genotyping

Genotyping of 91 SSR markers was done in two phases. A set of 49 markers were genotyped in Molecular Breeding Laboratory of Div. of Genetics and Plant Breeding, SKUAST-Jammu on 10% PAGE and another set of 42 markers were genotyped in Genotyping Centre at Center of Excellence in Genomics (CEG), ICRISAT-Hyderabad. At SKUAST-Jammu, the polymerase chain reaction (PCR) was done on thermal cycler (96 universal gradient, Peqlab, Germany) in 10 μl final volume containing 10–20 ng DNA template, 5.0 pmol forward and reverse primers, 1X PCR buffer, 2.5 mM of each dNTPs, 1.5mM MgCl_2_ and 0.5 U of Taq DNA polymerase (Himedia). The amplification conditions for PCR profile were: 95°C for 5 min, 40 cycles of 95°C for 1 min, specific annealing temperature for each SSR for 1 min, extension at 72°C for 1 min and final extension at 72°C for 10 min. The PCR products were visualized on 2% agarose gels for confirming amplification before being resolved on 10% Polyacrylamide gel electrophoresis (PAGE) using Dual Gel Vertical Electrophoresis System (Peqlab) followed by silver staining [24]. The scoring of marker alleles of SSRs was done manually using the stained gels.

The SSR genotyping at ICRISAT Hyderabad was done using capillary gel electrophoresis using ABI3700 automatic DNA sequencer. PCR for amplification of SSR loci were performed using thermal cycler GeneAmp PCR System 9700 (Applied Biosystems). The PCR reaction volume was 10 μl containing 5 ng DNA template, 10.0 pmol reverse and forward primers, 1X PCR buffer, 2.0mM of each dNTPs, 1.5mM MgCl_2_ and 0.3 U of Taq DNA polymerase. PCR amplification was done by touchdown profile with 3 min of initial denaturation cycle, followed by first five cycles of 94°C for 20 s, 60°C for 20 s and 72°C for 30 s, with 1°C decrease in annealing temperature per cycle, then 30 cycles of 94°C for 20 s with constant annealing temperature (56°C) and 72°C for 30 s followed by a final extension for 20 min at 72°C. The quality of amplified products was checked on 1.2% agarose gel. PCR amplified products were size fractioned using capillary electrophoresis on an ABI3700 automatic DNA sequencer (Applied Biosystems, USA). Allele sizing of the electrophoretic data was done using software Gene Mapper version 4 [25].

### Data analyses

The descriptive statistics from the trait data which includes range, mean, variance and frequency distribution was calculated using Microsoft Excel for both the field trial locations viz. SKUAST-Jammu and Bhaderwah, Jammu.

The identification of population structure and number of clusters in the 96 common bean lines was done using STRUCTURE software version 2.3.4 [26] which is a model-based clustering. The information obtained through model-based clustering (Q-matrix) was used to avoid false associations while working out marker–trait associations (MTAs).

Association mapping for identification of significant marker-trait associations for anthracnose was done using trait data on anthracnose from both locations and SSR marker data (91 SSRs) using software program TASSEL 3.0 (http://www.maizegenetics.net). Two different models were employed to calculate MTAs; general linear model (GLM) based on the Q-matrix derived from STRUCTURE software and mixed linear model (MLM) based on both the Q-matrix and the kinship matrix (K-matrix) derived from the marker data using TASSEL software. Significance of marker-trait associations were described as P-value (P = ≤ 0.05 for significant markers).

The P-values plots like −log10(P) genome-wide association plots (Manhattan plots) and quantile-quantile (QQ) have been used to for presentation of GWAS results using software program TASSEL. Manhattan plots represent the P values (y-axis) of all the markers used in genomic order by chromosome (x-axis).The QQ plot presents the graphical representation of the deviation of the observed P values from the null hypothesis: the observed P values for each SSR marker are sorted from largest to smallest and plotted against expected values from a theoretical χ 2 -distribution.

## Results and discussion

North-western Himalayan region that includes state Jammu and Kashmir possess huge diversity for common bean landraces [8]. Among various biotic stresses, Anthracnose is the most prevalent and devastating disease affecting bean production and productivity in this region. Therefore, developing common bean cultivars with resistance to anthracnose is considered one of the most effective ways of controlling this important disease [27]. Efforts have been made during the present study to characterize a set of 96 diverse bean lines selected from 428 lines for anthracnose resistance (for constitution of core set of 96 lines see [8]). Identification of sources of resistance as well as genetic dissection for anthracnose using genome-wide markers is considered one of the best approaches to discover useful marker-trait associations (MTAs) for this devastating disease. The major and stable MTAs once identified will prove useful for improvement of anthracnose resistance of local landraces of bean grown in this region through marker-assisted breeding programs.

### Trait evaluation

**Fig 1:**
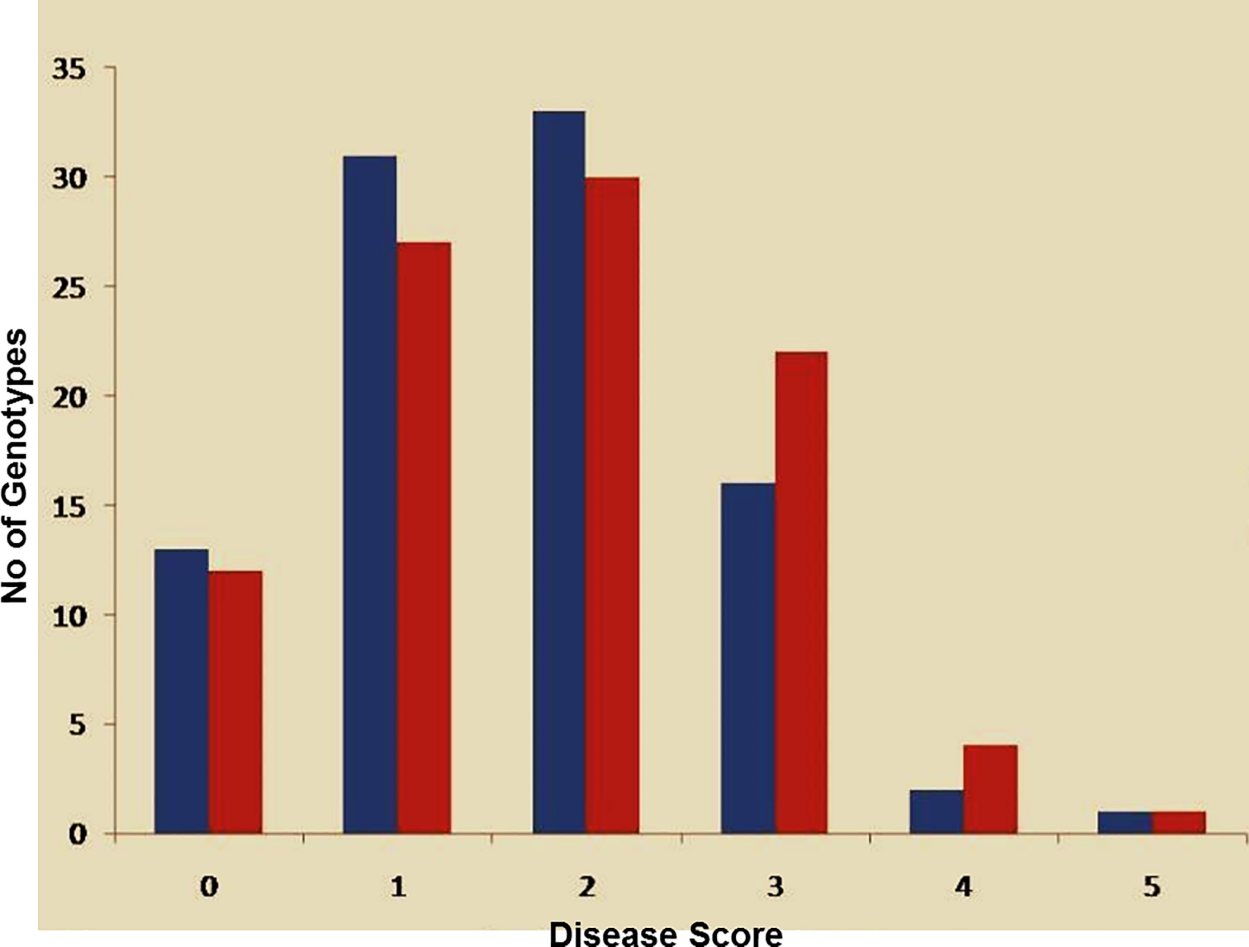
Frequency distribution of Anthracnose disease resistance score of core set of 96 bean lines. The blue color plots shows Anthracnose disease score recorded at SKUAST-Jammu while as red colour plots shows Anthracnose disease score recorded at Bhaderwah Jammu.

A set of 96 diverse bean lines comprising 45 Mesoamerican and 51 Andean were tested during the present study for anthracnose resistance at two locations. The analysis revealed that disease resistance of bean lines varied from 0 (anthracnose resistant) to 5 (anthracnose susceptible) across different locations (Fig 1). The mean value of anthracnose disease score was 1.65 at Jammu and 1.81 at Bhaderwah location. The sample variance recorded for Jammu location was 1.09 and 1.25 for Bhaderwah location. The 96 common bean lines in the present study were mainly categorized into three classes i.e., resistant (disease score 0 and 1), moderately susceptible (disease score 2 and 3) and susceptible lines (disease score 4 and 5). At SKUAST-Jammu location, 44 lines were declared resistant (19 Mesoamerican lines, 25 Andean), 49 moderately susceptible (26 Mesoamerican and 23 Andean) and 03 susceptible (Andean), while as at Bhaderwah location, 39 lines were found resistant (19 Mesoamerican and 20 Andean), 52 moderately susceptible (24 Mesoamerican and 28 Andean) and 05 susceptible (2 Mesoamerican and 3 Andean) to anthracnose. The distribution of disease score among 96 lines at both locations was skewed towards moderate susceptibility with around 50% of genotypes at both locations showing disease score of 2 and 3. The availability of different disease score for different lines in 96 core set for anthracnose resistance indicated its suitability for study of marker-trait associations for anthracnose resistance.

### Whole-genome SSR marker genotyping

A set of 91 SSR markers used during the present study belongs to all the 11 common bean chromosomes/linkage groups. The number of markers in an individual linkage group varied from 5 (LG10) to 14 (LG-02) with an average of 8.3 markers/chromosome. The 91 SSRs was judiciously selected based on earlier published reports (details in material and methods). Among 91 SSR markers, 90 SSRs were found polymorphic detecting more than 1 alleles/locus. The total number of alleles detected in core set was 691.The number of alleles varied from two (02) for SSR marker Bmr205 to 21 for SSR marker BM187 with an average of 7.59 alleles/locus. The allelic data was used for population structural analysis and working out marker-trait association (MTAs). The allelic diversity based on 91 SSR markers distributed throughout the bean genome indicated high diversity of core set and therefore suitability of germplasm for working out marker-trait associations for anthracnose.

### Marker-trait associations (MTAs) for anthracnose

**Fig 2:**
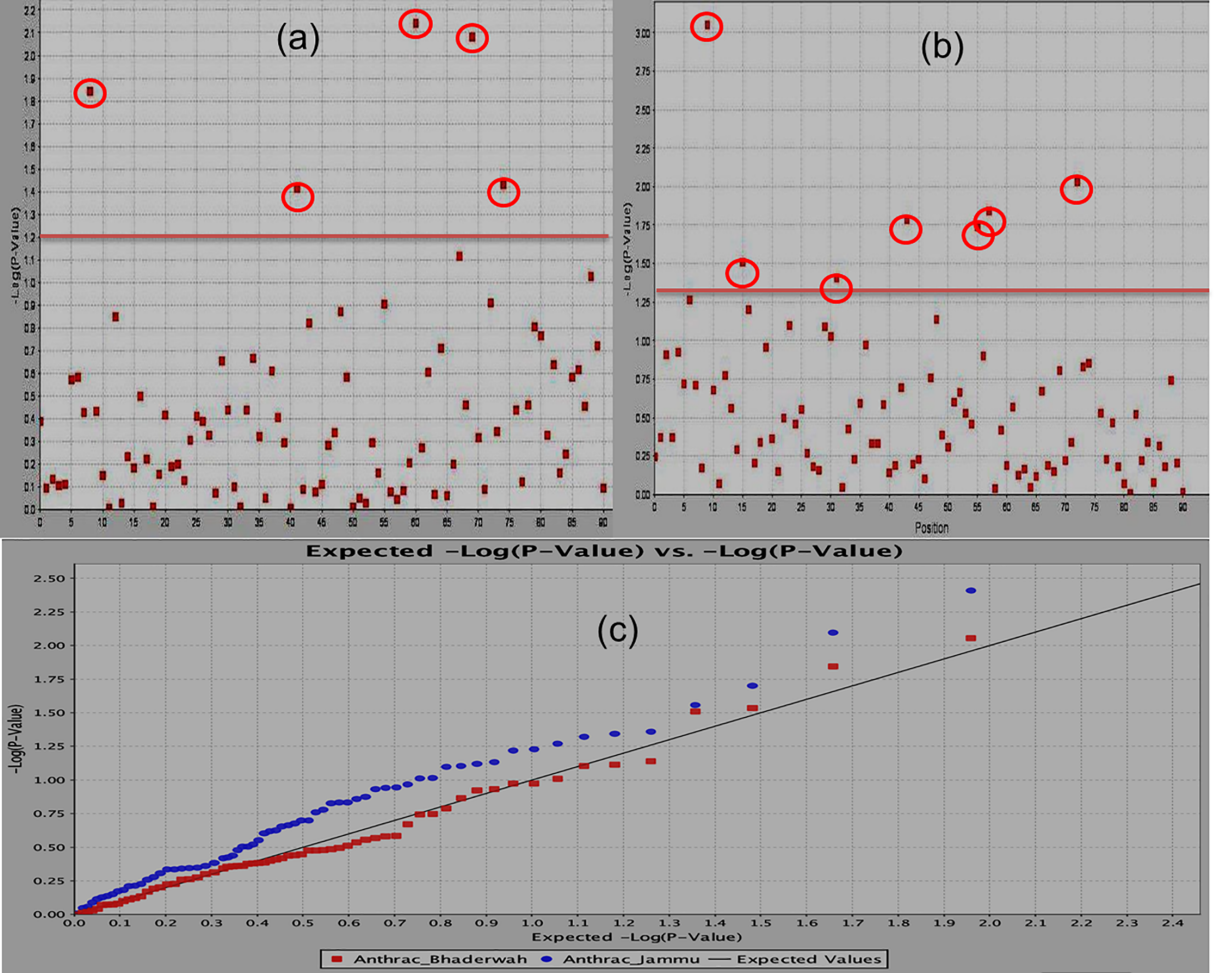
Manhattan plot showing significant MTAs identified using GLM approach of software program TASSEL for Anthracnose resistance. (a) Shows MTAs identified from the phenotypic data recorded at Bhaderwah-Jammu, (b) shows MTAs identified from the phenotypic data recorded at SKUAST- Jammu and (c) shows the QQ plots for both locations (SKUAST-Jammu and Bhaderwah Jammu.

The MTAs were identified by using both mixed linear model and general linear model of software program TASSEL. The MLM is considered better than GLM in controlling false positives but sometimes more stringent controls of MLM introduce false negatives and introduce missed heritability. So both models were employed to avoid any missed chance of heritability and identification of significant MTAs. The study of MTAs during the present study led to the identification of novel 10 MTAs (on chromosome number 1, 2, 4 and 8): 7 were identified by both GLM and MLM approaches, while as three (03) MTAs were identified by GLM approach only (Table 1; Fig 2). Among 10 MTAs, 7 were identified in Jammu environment, 5 in Bhaderwah environment and 5 in data pooled over environments (Fig 2). One MTA (Bmd45) was identified in all the three data sets and other MTAs were identified in either one or two environments (Table 1).

**Table 1:** Identification of new and validation of already know marker-trait associations for Anthracnose in common bean.

Marker	Chr.	Environment			P-value (Range)	R^2^ (%) (Range)	Method	Allele	Effect
		Bhaderwah, Jammu	SKUAST-Jammu	Pool					
**BM156**	2	**×**	**√**	**×**	0.01–0.04	13.0–16.0	GLM, MLM	288:288	-2.24456
**BM165**	8	**×**	**√**	**√**	0.007–0.04	13.3–17.7	GLM, MLM	193:193	-0.52561
**BM199**	4	**√**	**×**	**√**	0.02–0.04	16.9–18.6	GLM, MLM	296:304	-2.11537
**Bmd02**	2	**×**	**√**	**√**	0.009–0.04	10.3–13.6	GLM, MLM	121:121	-2.17985
**Bmd25**	8	**×**	**√**	**√**	0.02–0.03	16.0–17.1	GLM, MLM	124:124	3.922556
**Bmd45**	1	**√**	**√**	**√**	0.01–0.02	12.4–13.6	GLM, MLM	92:92	0.834771
**PVctt1**	4	**×**	**√**	**×**	0.008–0.03	21.3–22.0	GLM, MLM	177:177	3.178583
**BM211**	8	**√**	**√**	**×**	0.0008–0.04	18.0–25.45	GLM	270:270	0.78896
**PVBR251**	2	**√**	**×**	**×**	0.02	17.5	GLM	295:295	0.847005
**PVgaat001**	4	**√**	**×**	**×**	0.04	11.2	GLM	116:116	1.256582

The table presents 10 significant marker-trait associations for Anthracnose along with their chromosome number, environment of detection and phenotypic variation explained (PVE% or R^2^), method of detection (general linear model (GLM), mixed linear model (MLM), allele associated and allele effect.

The phenotypic variation explained (PVE%) by these MTAs varied from 10.3 (Bmd02) to 25.45 (BM211). Two MTAs (PVctt1 and BM211) are major explaining >20% phenotypic variation for anthracnose resistance and two MTAs (BM45 and BM211) are stable identified from both locations trait data. The major and stable MTAs are considered important and will be recommended for common bean molecular breeding programs aimed at enhancing anthracnose resistance of local bean landraces for north-western Himalayan state of Jammu and Kashmir.

A dozens of genes (*Co-1* to *Co-14*) have been already mapped for Anthracnose in common bean on different linkage groups. Genes specific for Mesoamerican gene pool and Andean gene pool have been also identified in earlier studies [28,29,30, 31]. For instance, three genes including *Co-1*, *Co-x* and *Co-w* have been mapped on Pv01 [32, 33], one gene *Co-u* g have been mapped on Pv02 [34] and six genes including *Co-3*, *Co-9*, *Co-y*, *Co-z*, *Co-10* and *Co-15* have been mapped on Pv04 for anthracnose resistance [33, 35, 36].

### Validation of genes/QTLs for anthracnose and important markers for marker-assisted selection (MAS)

**Fig 3:**
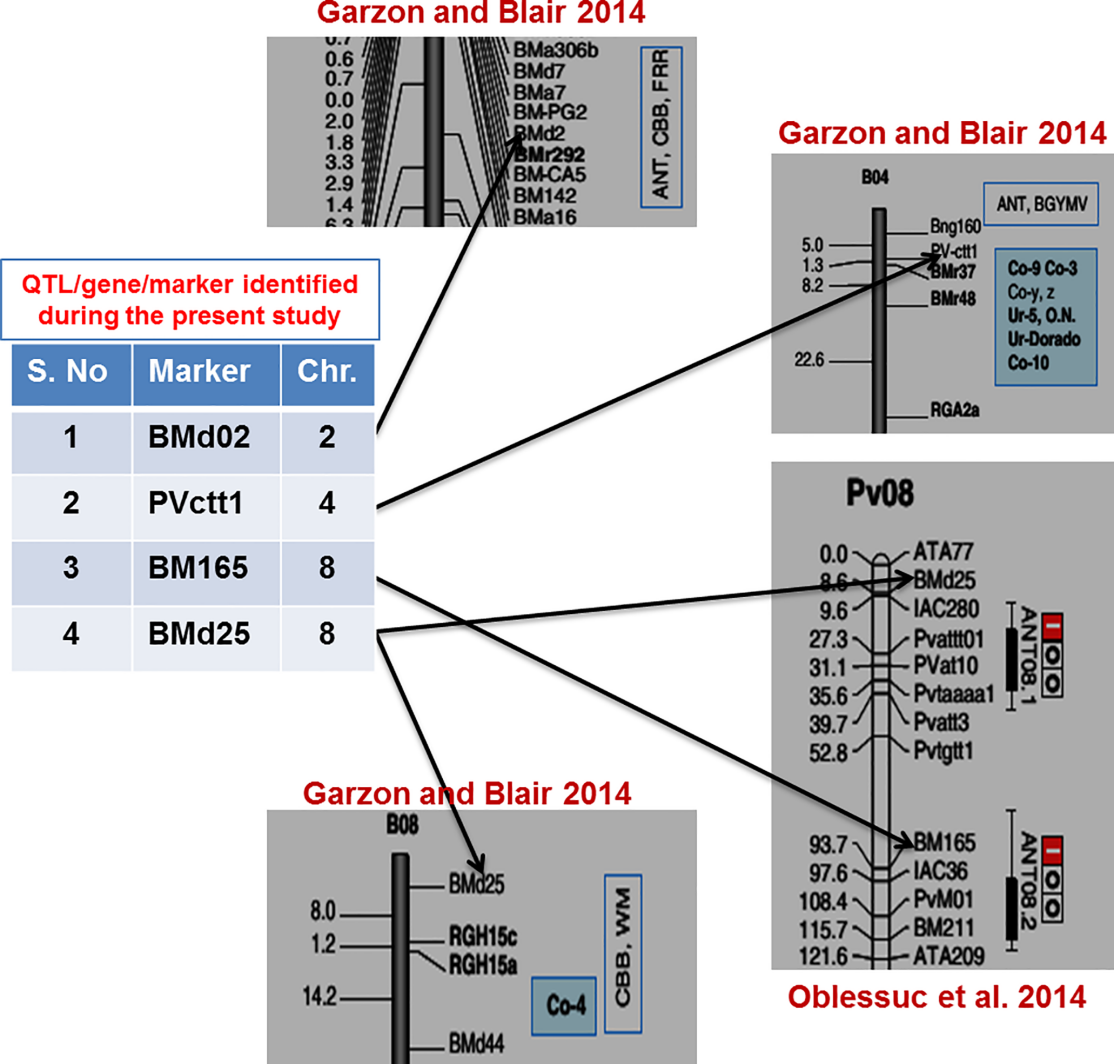
Validation of four QTLs/ Genes/markers for Anthracnose during the present study. The figure shows the location/map positions of four validated markers in/near the QTL/Genes clusters for Anthracnose identified in earlier studies/genetic linkage maps.

Among the 10 markers found associated with Anthracnose during the present study, a set of four markers (Bmd02, Pvctt1, BM165 and Bmd25) were found associated with the already identified QTLs/genes identified for Anthracnose [29, 30, 31] (Fig 3). The SSR marker Bmd02 was found in the vicinity of Anthracnose QTL demarcated on LG02 [31]. Therefore, the associated marker (Bmd02) identified during the present study for Anthracnose resistance may represent the same QTL regions as identified by Garzon and Blair [31]. Similarly, the associated marker Bmd25 represent the same genomic region harboring Anthracnose gene “*Co-4*” [31] and QTL for Anthracnose ANT08.1 [30]. The other associated marker BM165 on the chromosome PV08 represents the QTL region (*ANT08*.*2*) already identified for Anthracnose resistance [30]. The major MTA identified for Anthracnose during the present study (PVctt1) on PV04 lies in the same genomic region where a cluster of genes for Anthracnose (*Co-9*, *Co-3*, *Co-y*, *Co-z*, *Co-10*) have been placed in an earlier study [31]. Keeping this in view it is believed that these four markers found associated during the present study validated several genes/QTL identified in earlier studies. Therefore, these validated markers may provide good opportunity and are recommended for common bean molecular breeding programs for enhancing Anthracnose resistance of beans grown in north-western Himalayan region of state J&K, India. The validity of four MTA out of 10 MTAs identified during the present study confirmed the reliability of our results and therefore the new MTA that are identified for the first time (to the best of our knowledge) may prove useful for common bean breeding programs through MAS.

### Candidate lines for molecular breeding

Phenotypic evaluation of 96 common bean lines for anthracnose disease incidence at two locations viz SKUAST-Jammu and Bhaderwah, Jammu led to the identification of anthracnose resistant genotypes at both locations. Thirteen genotypes were found resistant for SKUAST-Jammu and twelve genotypes for SKUAST-Bhaderwah with anthracnose disease score 0. Only eight genotypes (PBG-4, PBG-6, PBG-13, PBG-17, PBG-19, PBG-24, PBG-31 & PBG-90; S1 Table) among these common bean lines were found stable across both locations with disease score “0”. Six genotypes among these were of exotic origin and two genotypes (PBG-31 & PBG-90) were local landraces. These genotypes can be utilized for future molecular breeding programs to improve anthracnose resistance in local landraces and cultivars of Jammu and Kashmir which are anthracnose susceptible but otherwise have good market value due to their colour, texture, taste and palatability. These lines can be also used in the development of bi-parental mapping populations, multi-parental populations and for differential gene expression studies.

## Supporting information

S1 Table Details of disease reaction of 96 bean lines towards *Colletotrichum lindemuthianum* inoculation at common bean hot-spot “Bhaderwah”. (DOCX)

## References

[1] Rodiño AP, Santalla M, González AM, De Ron AM, Singh SP. Novel genetic variation in common bean from the Iberian Peninsula. Crop Sci. 2006; 46: 2540–2546

[2] Singh N, Kaur S, Rana JC, Nakaura Y, Inouchi N. Isoamyl based branched fractions and granule size in starches from kidney bean germplasm: Distribution and relationship with functional properties. Food Res Int. 2012; 47: 174–181

[3] Rana JC, Sharma TR, Tyagi RK, Chahota RK, Gautam NK, Singh M, et al Characterisation of 4274 accessions of common bean (*Phaseolus vulgaris* L.) germplasm conserved in the Indian gene bank for phenological, morphological and agricultural traits. Euphytica. 2015; 205: 441–457

[4] Beebe S, Gonzalez AV, Rengifo J. Research on trace minerals in the common bean. Food Nutr Bull. 2000; 21: 387–391

[5] Broughton WJ, Hernandez G, Blair M, Beebe S, Gepts P, Vanderleyden J. Beans (*Phaseolus spp*.)–model food legumes. Plant Soil. 2003; 252: 55–128

[6] Santalla M, Fueyo MA, Rodino AP, Montero I, de Ron AM. Breeding for culinary and nutritional quality of common bean (*Phaseolus vulgaris* L.) in intercropping systems with maize (Zea mays L.). Biotechnologie, Agronomie, SociétéetEnvironnement. 1999; 3: 225–229

[7] FAO–Food and Agriculture Organization of the United Nations (2014) Food and agricultural commodities production. http://faostat.fao.org/site/339/default.aspx

[8] Choudhary N, Singh B, Khandy I, Sofi PA, Bhat MA, Mir RR. Insight into Common bean (*Phaseolus vulgaris* L.) Origin from North-Western Himalayas of State Jammu and Kashmir. Genet. Resour. Crop Evol. 2017 doi: 10.1007/s10722-017-0518-0

[9] Rodríguez‐Guerra R, Ramírez‐Rueda MT, La Vega D, Martínez O, Simpson J. Variation in genotype, pathotype and anastomosis groups of Colletotrichum lindemuthianum isolates from Mexico. Plant Pathol. 2003; 52: 228–235

[10] González-Chavira M, Guerra RR, Hernández-Godínez F, Acosta-Gallegos JA, de la Vega OM, Simpson J. Analysis of pathotypes of Colletotrichum lindemuthianum found in the central region of Mexico and resistance in elite germplasm of *Phaseolus vulgaris*. Plant Dis. 2004; 88: 152–156 10.1094/PDIS.2004.88.2.152 30812421

[11] Sharma PN, Padder BA, Sharma OP, Pathania A, Sharma P. Pathological and molecular diversity in *Colletotrichum lindemuthianum* (bean anthracnose) across Himachal Pradesh, a north-western Himalayan state of India. Australas. Plant Pathol. 2007; 36: 191–197

[12] Melotto M, Balardin RS, Kelly JD. Host-pathogen interaction and variability of *Colletotrichum lindemuthianum* *Colletotrichum* host specificity, pathology, host–pathogen interaction. APS press, St. Paul, MN 2000; pp346–361

[13] Fernández MT, Fernandez M, Casares A, Rodriguez R, Fueyo M. Bean germplasm evaluation for anthracnose resistance and characterization of agronomic traits: A new physiological strain of *Colletotrichum lindemuthianum* infecting Phaseolus vulgaris L. in Spain. Euphytica. 2000; 114: 143–149

[14] Miklas PN, Kelly JD, Beebe SE, Blair MW. Common bean breeding for resistance against biotic and abiotic stresses: from classical to MAS breeding. Euphytica. 2006; 147: 105–131

[15] Drijfhout E, Davis JH. Selection of a new set of homogeneously reacting bean (Phaseolus vulgaris) differentials to differentiate races of *Colletotrichum lindemuthianum*. Plant Pathol. 1989; 38: 391–396

[16] Gaitán-Solís E, Duque MC, Edwards KJ, Tohme J. Microsatellite Repeats in Common Bean. Crop Sci. 2002; 42: 2128–2136

[17] Yu K, Park SJ, Poysa V, Gepts P. Integration of simple sequence repeat (SSR) markers into a molecular linkage map of common bean (*Phaseolus vulgaris* L.). J. Hered. 2000; 91: 429–434 11218079 10.1093/jhered/91.6.429

[18] Blair MW, Pedraza F, Buendia HF, Gaitán-Solís E, Beebe SE, Gepts P, et al Development of a genome-wide anchored microsatellite map for common bean (*Phaseolus vulgaris* L.). Theor. Appl. Genet. 2003; 107: 1362–1374 doi: 10.1007/s00122-003-1398-6 14504741 10.1007/s00122-003-1398-6

[19] Blair MW, Giraldo MC, Buendia HF, Tovar E, Duque MC, Beebe SE. Microsatellite marker diversity in common bean (*Phaseolus vulgaris* L.). Theor. Appl. Genet. 2006; 113: 100–109 doi: 10.1007/s00122-006-0276-4 16614831 10.1007/s00122-006-0276-4

[20] Blair MW, González LF, Kimani PM, Butare L. Genetic diversity, inter-gene pool introgression and nutritional quality of common beans (*Phaseolus vulgaris* L.) from Central Africa. Theor. Appl. Genet. 2010; 121: 237–248 doi: 10.1007/s00122-010-1305-x 20224891 10.1007/s00122-010-1305-x PMC2886139

[21] Grisi MC, Blair MW, Gepts P, Brondani C, Pereira PA, Brondani RP. Genetic mapping of a new set of microsatellite markers in a reference common bean (*Phaseolus vulgaris*) population BAT93 x Jalo EEP558. Genet. Mol. Res. 2007; 6: 691–706 18050090

[22] Galeano CH, Cortés AJ, Fernández AC, Soler Á, Franco-Herrera N, Makunde G, Vanderleyden J, Blair MW. Gene-based single nucleotide polymorphism markers for genetic and association mapping in common bean. BMC genetics. 2012; 13: 48 doi: 10.1186/1471-2156-13-48 22734675 10.1186/1471-2156-13-48 PMC3464600

[23] Garzon LN, Blair MW. Development and mapping of SSR markers linked to resistance-gene homologue clusters in common bean. Crop J. 2014; 2: 183–194

[24] Tegelstrom H. Detection of mitochondrial DNA fragments Molecular genetic analysis of populations: a practical approach. IRL Press, Oxford 1992; 89–114

[25] Chatterji S, Pachter L. Reference based annotation with GeneMapper. Genome Biol. 2006; 7: R29 doi: 10.1186/gb-2006-7-4-r29 16600017 10.1186/gb-2006-7-4-r29 PMC1557983

[26] Pritchard JK, Stephens M, Donnelly P. Inference of population structure using multilocus genotype data. Genetics. 2000; 155: 945–959 10835412 10.1093/genetics/155.2.945 PMC1461096

[27] Zuiderveen GH, Padder BA, Kamfwa K, Song Q, Kelly JD. Genome-wide association study of anthracnose resistance in andean beans (*Phaseolus vulgaris*). PloS ONE. 2016; 11: e0156391 doi: 10.1371/journal.pone.0156391 27270627 10.1371/journal.pone.0156391 PMC4894742

[28] Kelly JD, Vallejo VA. A comprehensive review of the major genes conditioning resistance to anthracnose in common bean. Hort. Sci.2004; 39:1196–1207

[29] Campa A, Rodríguez-Suárez C, Giraldez R, Ferreira JJ. Genetic analysis of the response to eleven *Colletotrichum lindemuthianumraces* in a RIL population of common bean (*Phaseolus vulgaris* L.). BMC Plant Biol 2014; 14:115 doi: 10.1186/1471-2229-14-115 24779442 10.1186/1471-2229-14-115 PMC4021056

[30] Oblessuc PR, Baroni RM, da Silva Pereira G, Chiorato AF, Carbonell SAM, Brin˜ez B, et al Quantitative analysis of race specific resistance to *Colletotrichum lindemuthianumin* common bean. Mol Breeding 2014; 34:1313–1329

[31] Garzon LN, Blair MW. Development and mapping of SSR markers linked to resistance-gene homologue clusters in common bean. The Crop Journal 2014; 2: 183–194

[32] Geffroy V, Sévignac M, Billant P, Dron M, Langin T. Resistance to *Colletotrichum lindemuthianum* in Phaseolus vulgaris: a case study for mapping two independent genes. Theor. Appl. Genet. 2008; 116: 407–415 doi: 10.1007/s00122-007-0678-y 18060540 10.1007/s00122-007-0678-y

[33] Rodríguez-Suárez C, Ferreira JJ, Campa A, Pañeda A, Giraldez R. Molecular mapping and intra-cluster recombination between anthracnose race-specific resistance genes in the common bean differential cultivars Mexico 222 and Widusa. Theor. Appl. Genet. 2008; 116: 807–814 doi: 10.1007/s00122-008-0714-6 18210079 10.1007/s00122-008-0714-6

[34] Geffroy V, Macadré C, David P, Pedrosa-Harand A, Sévignac M, Dauga C, Langin T. Molecular analysis of a large subtelomeric nucleotide-binding-site–leucine-rich-repeat family in two representative genotypes of the major gene pools of *Phaseolus vulgaris*. Genetics. 2009181: 405–419 10.1534/genetics.108.093583 PMC2644936 19087965

[35] Alzate-Marin AL, Costa MR, Arruda KM, De Barros EG, Moreira MA. Characterization of the anthracnose resistance gene present in Ouro Negro (Honduras 35) common bean cultivar. Euphytica. 2003; 133: 165–169

[36] Sousa LL, Gonçalves-Vidigal MC, Gonçalves AO, VidigalFilho PS, Awale H, Kelly JD. Molecular mapping of the anthracnose resistance gene *Co-15* in the common bean cultivars Corinthiano. Ann Rep Bean Improv Coop. 2013; 56: 45–46

