# Supplementary material for: Gene/QTL discovery for Anthracnose in common bean (Phaseolus vulgaris L.) from North-western Himalayas
Source: PLoS One. 2018 Feb 1;13(2):e0191700. doi: 10.1371/journal.pone.0191700 (PMC5794095; doi:10.1371/journal.pone.0191700)
Supplement: S1 Table — (DOCX) [file pone.0191700.s001.docx]

**S1 Table.**

| S. No. | Genotype | Pedigree | Disease Reaction |
| --- | --- | --- | --- |
| 1 | PBG-01 | Exotic | Moderately susceptible |
| 2 | PBG-02 | Exotic | Moderately susceptible |
| 3 | PBG-03 | Exotic | Resistant |
| 4 | PBG-04 | Exotic | Resistant |
| 5 | PBG-05 | Exotic | Resistant |
| 6 | PBG-06 | Exotic | Resistant |
| 7 | PBG-07 | Exotic | Moderately susceptible |
| 8 | PBG-08 | Exotic | Resistant |
| 9 | PBG-09 | Exotic | Moderately susceptible |
| 10 | PBG-10 | Exotic | Resistant |
| 11 | PBG-11 | Indigenous/Kashmir | Resistant |
| 12 | PBG-12 | Indigenous/Kashmir | Moderately susceptible |
| 13 | PBG-13 | Exotic | Resistant |
| 14 | PBG-14 | Exotic | Resistant |
| 15 | PBG-15 | Exotic | Moderately susceptible |
| 16 | PBG-16 | Exotic | Moderately susceptible |
| 17 | PBG-17 | Exotic | Resistant |
| 18 | PBG-18 | Indigenous/Kashmir | Moderately susceptible |
| 19 | PBG-19 | Exotic | Resistant |
| 20 | PBG-20 | Exotic | Susceptible |
| 21 | PBG-21 | Exotic | Resistant |
| 22 | PBG-22 | Exotic | Resistant |
| 23 | PBG-23 | Indigenous/Kashmir | Susceptible |
| 24 | PBG-24 | Exotic | Resistant |
| 25 | PBG-25 | Exotic | Resistant |
| 26 | PBG-26 | Indigenous/Kashmir | Moderately susceptible |
| 27 | PBG-27 | Indigenous/Kashmir | Moderately susceptible |
| 28 | PBG-28 | Indigenous/Jammu | Resistant |
| 29 | PBG-29 | Indigenous/Kashmir | Resistant |
| 30 | PBG-30 | Indigenous/Kashmir | Moderately susceptible |
| 31 | PBG-31 | Indigenous/Kashmir | Resistant |
| 32 | PBG-32 | Indigenous/Kashmir | Moderately susceptible |
| 33 | PBG-33 | Indigenous/Jammu | Moderately susceptible |
| 34 | PBG-34 | Indigenous/Kashmir | Moderately susceptible |
| 35 | PBG-35 | Indigenous/Jammu | Moderately susceptible |
| 36 | PBG-36 | Indigenous/Jammu | Moderately susceptible |
| 37 | PBG-37 | Indigenous/Jammu | Moderately susceptible |
| 38 | PBG-38 | Indigenous/Jammu | Moderately susceptible |
| 39 | PBG-39 | Indigenous/Kashmir | Moderately susceptible |
| 40 | PBG-40 | Indigenous/Kashmir | Resistant |
| 41 | PBG-41 | Indigenous/Kashmir | Moderately susceptible |
| 42 | PBG-42 | Varanasi/India | Moderately susceptible |
| 43 | PBG-43 | Indigenous/Kashmir | Moderately susceptible |
| 44 | PBG-44 | Indigenous/Kashmir | Moderately susceptible |
| 45 | PBG-45 | Indigenous/Kashmir | Moderately susceptible |
| 46 | PBG-46 | Indigenous/Kashmir | Resistant |
| 47 | PBG-47 | Indigenous/Kashmir | Resistant |
| 48 | PBG-48 | Exotic | Moderately susceptible |
| 49 | PBG-49 | Indigenous/Kashmir | Moderately susceptible |
| 50 | PBG-50 | Indigenous/Kashmir | Moderately susceptible |
| 51 | PBG-51 | Indigenous/Kashmir | Resistant |
| 52 | PBG-52 | Indigenous/Kashmir | Resistant |
| 53 | PBG-53 | Indigenous/Kashmir | Moderately susceptible |
| 54 | PBG-54 | Indigenous/Kashmir | Moderately susceptible |
| 55 | PBG-55 | Indigenous/Kashmir | Moderately susceptible |
| 56 | PBG-56 | Indigenous/Jammu | Moderately susceptible |
| 57 | PBG-57 | Exotic | Moderately susceptible |
| 58 | PBG-58 | Exotic | Susceptible |
| 59 | PBG-59 | Exotic | Moderately susceptible |
| 60 | PBG-60 | Exotic | Moderately susceptible |
| 61 | PBG-61 | Exotic | Resistant |
| 62 | PBG-62 | Exotic | Resistant |
| 63 | PBG-63 | Exotic | Moderately susceptible |
| 64 | PBG-64 | Indigenous/Kashmir | Resistant |
| 65 | PBG-65 | Indigenous/Kashmir | Moderately susceptible |
| 66 | PBG-66 | Exotic | Resistant |
| 67 | PBG-67 | Exotic | Moderately susceptible |
| 68 | PBG-68 | Exotic | Moderately susceptible |
| 69 | PBG-69 | Exotic | Moderately susceptible |
| 70 | PBG-70 | Exotic | Susceptible |
| 71 | PBG-71 | Exotic | Resistant |
| 72 | PBG-72 | Exotic | Moderately susceptible |
| 73 | PBG-73 | Exotic | Moderately susceptible |
| 74 | PBG-74 | Exotic | Moderately susceptible |
| 75 | PBG-75 | Exotic | Moderately susceptible |
| 76 | PBG-76 | Exotic | Moderately susceptible |
| 77 | PBG-77 | Exotic | Moderately susceptible |
| 78 | PBG-78 | Exotic | Moderately susceptible |
| 79 | PBG-79 | Indigenous/Kashmir | Moderately susceptible |
| 80 | PBG-80 | Indigenous/Kashmir | Resistant |
| 81 | PBG-81 | Indigenous/Kashmir | Resistant |
| 82 | PBG-82 | Indigenous/Kashmir | Resistant |
| 83 | PBG-83 | Indigenous/Kashmir | Moderately susceptible |
| 84 | PBG-84 | Indigenous/Kashmir | Resistant |
| 85 | PBG-85 | Indigenous/Kashmir | Resistant |
| 86 | PBG-86 | Indigenous/Kashmir | Moderately susceptible |
| 87 | PBG-87 | Indigenous/Kashmir | Resistant |
| 88 | PBG-88 | Indigenous/Kashmir | Resistant |
| 89 | PBG-89 | Indigenous/Kashmir | Resistant |
| 90 | PBG-90 | Indigenous/Kashmir | Resistant |
| 91 | PBG-91 | Indigenous/Kashmir | Susceptible |
| 92 | PBG-92 | Indigenous/Kashmir | Moderately susceptible |
| 93 | PBG-93 | Indigenous/Kashmir | Moderately susceptible |
| 94 | PBG-94 | Indigenous/Kashmir | Resistant |
| 95 | PBG-95 | Indigenous/Kashmir | Moderately susceptible |
| 96 | PBG-96 | Indigenous/Kashmir | Resistant |
